# Sunshine and Shadow

**Published:** 1893-07

**Authors:** 


### Editorial.

### SUNSHINE AND SHADOW.

In these early summer days the mind instinctively turns to the green fields and the purer air laden with the aroma of June roses and longs for the restful quiet denied in the rush and tumult of city life.

The demand for change, for new scenes, and an annual absorption of experiences other than those which the routine life of the year has given, is a part of our being, and should be accepted as necessary to it and adding to our capacity for more extended and healthful labor.

The sedentary life of our profession is not by any means the least of the evils connected with its practice. Hour by hour the dentist is placed in unhealthful conditions, day by day he faces the same routine of work, the same exacting fears of those under his care, feels the same nervous exhaustion from over-labor and the still more unendurable reflected nervous restlessness of patients, until nature rebels and he is forced to lay down the excavator and plugger and seek a rest denied him in his immediate surroundings.

The practice of dentistry is beyond question one of the most laborious and destructive to health of all the professions. The vitiated atmosphere, the constrained positions, the nervous strain, the unnoted pressure of the chair upon the body, resulting oftentimes in serious lesions, the monotony of practice,—all combined render it difficult and unsatisfactory if the reason be not allowed full supremacy in governing it.

On the other hand, if the operator will discard the idea of wealth and be satisfied with a reasonable competency, shorten the hours of labor, lessen the strain, admit the sunlight to his apartment, avoid mere routine practice, and make every operation a study and means of mental growth and advancement in scientific knowledge, it becomes the most agreeable and fascinating of employments.

The mistake made by many men is that they become not the willing servitor of patients, but their slave. They permit dictation of hours and yield moments to urgent request that should be devoted to physical recuperation. The warning that always comes from an overstrained nervous system goes unheeded until the final breakdown, when perhaps it may be too late; the verdict is rendered, sentence pronounced, and the individual leaves the ranks forever.

It would be interesting and very instructive if those who have lived longest in the practice of dentistry and maintained a fair share of health to old age could give their experience. Unfortunately, one by one these are passing away and have left no word of instruction or warning to younger men. The record of death of the latter has always been large in our ranks, and, in the experience of the writer, it has invariably been of those who have failed to remember that there is a limit to human endurance. We have often sounded the warning into unwilling ears, and have stood as often by the open casket with the vain regret that the advice was not heeded.

The hours of work in dentistry are too long. As practice grows the eager youth adds on to these until, in many cases, they extend far into the night. The strain of an eight-hour period is sufficient for any toiler, whether that be over the operating-chair or at other labor. Change of occupation is necessary both in manual and mental work, and when these are combined, as in our practice, it becomes imperative. It is said of Bancroft, the historian, that he would take an intermission in his labor and have a novel read to him. This was not only a rest, but infused new life into his imagination. It is trite to urge this alternation of work. All understand it, but few adopt it.

It is asserted by bacteriologists that sunshine is deadly to all micro-organisms. If this be true, how dangerous it may prove to remain day after day in the shadow of a northern light, and exchange this only for the heated gas of the laboratory!

It will be found, we think, that those who have lived the longest and enjoyed life the most have so arranged their periods of labor that the sunshine and shadow will each have had its due proportion.

To accomplish this means an orderly arrangement of professional labor with exercise in the sunshine, if possible, or at least out of doors. One of the most successful of practitioners in the writer's knowledge was one who invariably after a light breakfast started off for a walk of two miles in the parks adjacent to his city. He made this not a mere tramp, without an object, but sought entertainment and mental relaxation in the study of natural objects. A favorite method was to improve the surroundings by introducing rare trees from their foreign habitat and studying them as they developed in their new surroundings. He stocked a pond with goldfish, and it was interesting to watch the crowding of these golden beauties towards the shore as their benefactor walked to the edge, certain as they were of a dainty morsel from his hand. He returned from this exercise invigorated for a day's work, and at five in the afternoon he was off again for a ride or a walk, preferably the latter.

The combination of sunshine and shadow, play and labor, must come into the life of every operator if health is to be maintained. In the writer's opinion there should be a break every day at twelve o'clock, a light lunch, followed by a walk in the noonday sun. The return to work at one will be with body refreshed and the mental forces strengthened.

The invariable response to this is, "I cannot break away; patients are pressing, and it means just so many dollars from my income." Patients should be trained to know that hours of relaxation are not to be infringed upon, and in the end the money gained, if gained at all, is eventually lost in broken health.

The traveller in European lands is surprised to find in many places that the idea here sought to be conveyed has become the settled habit of the people. The work of the world seems to have come to a stand-still at the noon-hour. The money-changer leaves his temple, the merchant the mart of trade, and an intermission of two hours' rest is taken, feeding the wants of the body and refreshing the mind. Whether this be the true idea or not, it is certainly better than the continued rush adopted in this country, and it is especially desirable that in our profession there should be a change made by the partial adoption of this mode of division of the day's work.

The proper commingling of the dynamic forces of the great luminary with the no less potent change of occupation should bring sunshine and health into every dentist's experience. We cannot measure the molecular forces in the ever-changing human organism,—the tendency here to disease and there to health,—but we can by persistent activity and intelligent observation so regulate our habits that as dentists we can enjoy equally with others a fair measure of health and a minimum of discomfort in the practice of our profession.

